# Aqueous Two-Phase Systems within Selectively Permeable Vesicles

**DOI:** 10.1021/acsmacrolett.3c00341

**Published:** 2023-07-27

**Authors:** Berta Tinao, Juan L. Aragones, Laura R. Arriaga

**Affiliations:** Department of Theoretical Condensed Matter Physics, Condensed Matter Physics Center (IFIMAC) and Instituto Nicolás Cabrera, Universidad Autónoma de Madrid, 28049 Madrid, Spain

## Abstract

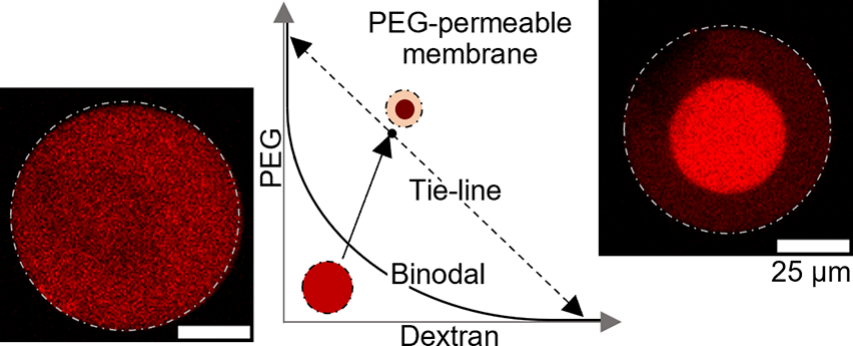

An aqueous two-phase system (ATPS) encapsulated within a vesicle organizes the vesicle core as two coexisting phases that partition encapsulated solutes. Here, we use microfluidic technologies to produce vesicles that efficiently encapsulate mixtures of macromolecules, providing a versatile platform to determine the phase behavior of ATPSs. Moreover, we use compartmentalized vesicles to investigate how membrane permeability affects the dynamics of the encapsulated ATPS. Designing a membrane selectively permeable to one of the components of the ATPS, we show that out-of-equilibrium phase separations formed by a rapid outflow of water can be spontaneously reversed by a slower outflow of the permeating component across the vesicle membrane. This dynamics may be exploited advantageously by cells to separate and connect metabolic and signaling routes within their nucleoplasm or cytoplasm depending on external conditions.

The phase behavior of aqueous mixtures of macromolecules is important in many practical applications, including the separation of biomolecules, organelles, and cells, as they often selectively partition into one of the coexisting phases, and the performance of bioconversions with simultaneous removal of products, thereby improving reaction yields.^1,2^ Phase separation also plays a vital role in a variety of cellular processes^3,4^ and provides a new framework for understanding the functional role of protein disorder within the cell cytoplasm.^5^ Cells control intracellular phase separations by controlling the concentration and distribution of macromolecules in their cytoplasm.^5^ To maintain, create, or reverse phase separations, the cell is capable of producing local synthesis or degradation of macromolecules^3^ or transporting macromolecules across membranes.^6^ Synthetic models mimicking such complexity may help understand the dynamical organization of the cell cytoplasm.^7−9^ In this context, vesicles encapsulating aqueous two-phase systems are used to model intracellular organization and to partition molecules across the phases.^10^ Because an ATPS is formed from an aqueous mixture of sufficiently dissimilar macromolecules above a concentration threshold that depends on temperature, heating of the aqueous mixture provides an efficient route to reverse a phase separation.^11^ Alternatively, because vesicle membranes, much like cell membranes, are permeable to water, osmosis provides another route to influence phase separations and solute partitioning across different phases.^12−16^ Unfortunately, conventional methods to vesicle fabrication result in an uncontrolled encapsulation of macromolecules within the vesicles,^11^ which limits the utility of current vesicle models for quantitative studies on the phase behavior of ATPSs. The control encapsulation enabled by droplet-based microfluidic technologies overcomes the encapsulation limitations of conventional methods, enabling the formation of multiphase water-in-oil (W/O) droplets with specific phase compositions and thus solute partitioning.^17−21^ However, a multiphase W/O drop poorly mimics the rich dynamics and phase behavior that arise in the cell as a result of having a membrane separating the internal and external aqueous phases. It is therefore essential to achieve control over the encapsulation of ATPSs in vesicles to dynamically control phase separations. This can be achieved by using W/O/W double emulsion drops as vesicle templates.^22^ The control over the encapsulation of this approach may enable the use of vesicles to rapidly determine the phase behavior of ATPSs. Moreover, vesicles with selectively permeable membranes fabricated by microfluidics may enable us to demonstrate alternative routes to temperature and osmosis to control solute partitioning across coexisting phases. These vesicles may exploit the selective transport of certain macromolecules across the membrane, much like eukaryotes use transport systems spanning the nuclear membrane to selectively transport RNA and proteins from the nucleoplasm to the cytoplasm, and vice versa.^6^

In this work, we demonstrate the controlled encapsulation of an ATPS within vesicles using droplet microfluidics. The vesicle membrane is designed as a selectively permeable membrane, enabling the transport of one of the macromolecular components of the ATPS across the membrane. This design enables us to study the effect of a source/sink system, implemented as a large volume excess of the permeating macromolecule outside the vesicle, on the dynamics of phase separation of an ATPS encapsulated within the vesicles. In particular, we encapsulate mixtures of poly(ethylene glycol) (PEG, 6 kDa) and dextran (70 kDa), which phase separate above a certain concentration, within membranes made of a triblock copolymer of poly(ethylene glycol)–poly(propylene glycol)–poly(ethylene glycol) (PEG–PPG–PEG, Pluronic L121, 4.4 kDa), which is selectively permeable to PEG and favorably wetted by the PEG-rich phase. To study the effect of macromolecular transport on the dynamics of phase separation, compartmentalized vesicles with a dextran-rich core and a PEG-rich shell are exposed to a large volume excess of an aqueous solution with increasing PEG concentration. We observe the spontaneous reversal of phase separations at long times depending on external conditions; this opens the avenue to develop materials that sense and respond to changes in the environment. Moreover, after equilibration, these vesicles provide a platform to determine the phase behavior of the encapsulated system, which is important for biotechnology applications.

To produce polymer vesicles or polymersomes, we use W/O/W double emulsion drops with ultrathin middle oil layers as templates. These ultrathin shelled double emulsions are fabricated with a coflow glass-capillary microfluidic device,^23^ as further detailed in Section S1 of the Supporting Information. The inner water phase consists of a homogeneous polymer mixture of PEG and dextran, at a total polymer concentration of 5% (w/v; mass of solute per volume of solution) and varying PEG/dextran ratio. The middle oil phase contains 20% (w/v) Pluronic L121 dissolved in a mixture of 36% (v/v) chloroform and 64% (v/v) hexane. The outer phase is a 10% (w/v) poly(vinyl alcohol) (PVA, 13–23 kDa) aqueous solution. After production, the double emulsion drops are collected in vials containing a large volume excess of an aqueous solution of PEG (6 kDa) at concentrations ranging from 7 to 12% (w/v). Because the inner water phase encapsulated within the double emulsion drops is denser than the collection aqueous medium, double emulsion drops sediment, which facilitates their visualization on an inverted optical microscope. Moreover, these templates spontaneously yield vesicles upon removal of the middle phase solvent^24^ because Pluronic L121 is an amphiphilic polymer capable of forming vesicles.^25−28^ Upon dewetting of the middle phase solvent,^29−31^ the two Pluronic monolayers adsorbed at the O/W interfaces of the double emulsions self-assemble into a membrane. The final structure of this Pluronic membrane might be a mixture of folded polymers with the PEG blocks protruding out from either the same side of the membrane or both sides, given that the reported estimated membrane thickness is about five times smaller than the estimated length of a completely stretched hydrophobic PPG block,^25^ as illustrated schematically in Figure 1a. A typical confocal fluorescence image of the resultant vesicles and an amplified image of a single vesicle are shown in Figure 1b,c. However, the oil drop formed by dewetting remains attached to a membrane region, as illustrated schematically in the middle panel of Figure 1a, and upon evaporation of the remaining solvent, we typically observe an aggregated polymer patch in a certain membrane region, as illustrated in the rightmost panel of Figure 1a and shown by the white arrow in the vertical projection of the vesicle in Figure 1d. The typical distribution in both radius and inner mean fluorescence intensity of vesicles within a batch is exemplified in Figure 1e, which highlights the monodispersity in size and composition in this type of production.

**Figure 1:**
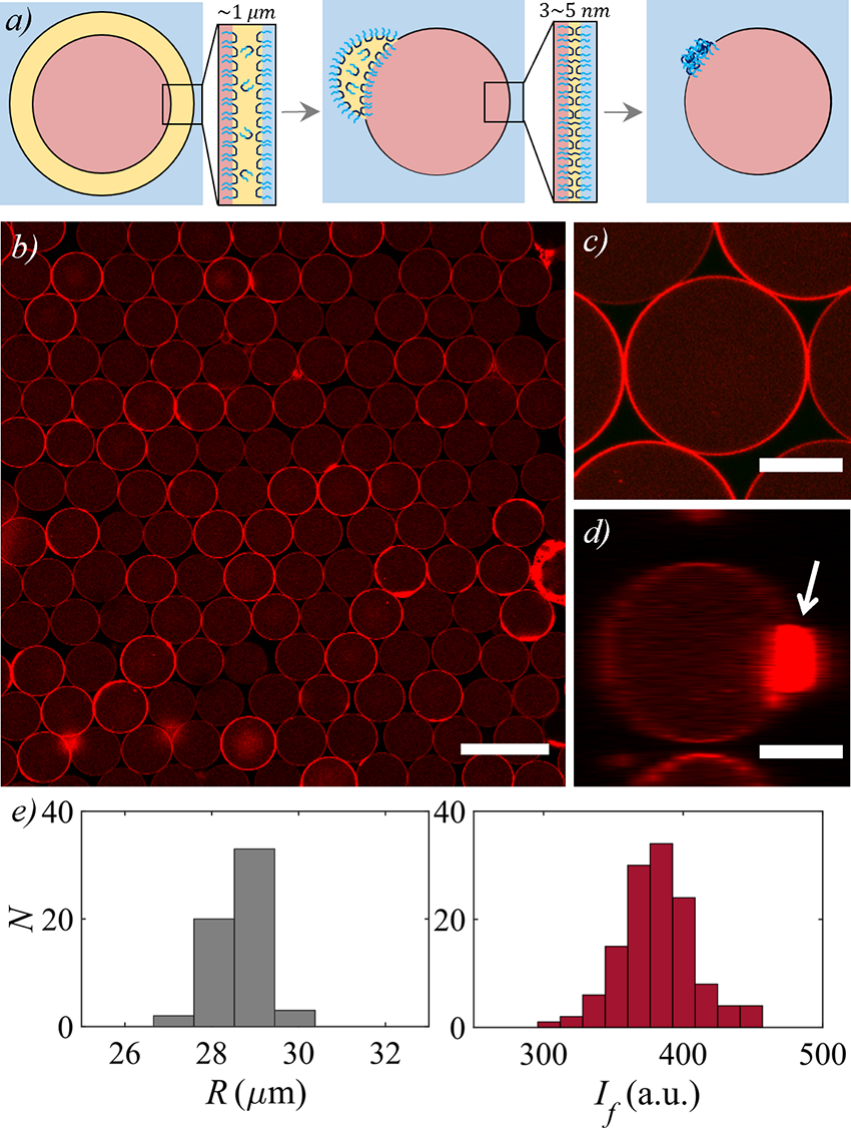
(a) Schematic illustration of the evolution of a double emulsion drop (left) into a polymer vesicle by dewetting (middle) and further evaporation of the middle phase solvent (right). (b) Confocal fluorescence microscopy image showing a typical production of vesicles containing an aqueous mixture of 2.5% (w/v) PEG and 2.5% (w/v) dextran, collected in an aqueous solution of 7% (w/v) PEG, with their membrane and aqueous cores fluorescently labeled with DOPE-Rh and dextran-Rh, respectively. Scale bar is 100 μm. (c) Detail of the equatorial plane of a vesicle. Scale bar is 25 μm. (d) Vertical projection of the vesicle, exhibiting an aggregate of polymer attached to the membrane, indicated by the white arrow. Scale bar is 25 μm. (e) Distribution of the radius (left) and mean fluorescence intensity of the aqueous cores of the vesicles (right) for the production shown in (b).

To create compartments within the inner cores of the vesicles, we concentrate the encapsulated polymer mixture subjecting the vesicles to an osmotic pressure difference.^12,14,16^ The osmotic pressure difference is imposed by collecting the vesicles in aqueous solutions of PEG with osmolarity higher than that of the aqueous polymer mixtures encapsulated in the inner vesicle cores. A water outflow then concentrates the polymer mixture yielding compartmentalized vesicles, as exemplified in Figure 2a. The dextran-rich compartment, labeled in red, is located toward the bottom center of the vesicles, while the PEG-rich phase surrounds the dextran-rich compartment and contacts the membrane, as shown in the vertical projections of Figure 2a. This core–shell configuration is enabled by both the chemical nature of Pluronic L121 that favors wetting of the membrane by the PEG-rich phase and the differences in density between both phases that makes the dextran-rich compartment sink. In this collection medium, both the vesicle and dextran-rich compartment remain spherical, as shown in the vertical projections in the top panel of Figure 2a, which enables us to calculate the volume of each phase once thermodynamic equilibrium is achieved from their measured radius. To ensure thermodynamic equilibrium, images are acquired 2 days after production.

**Figure 2:**
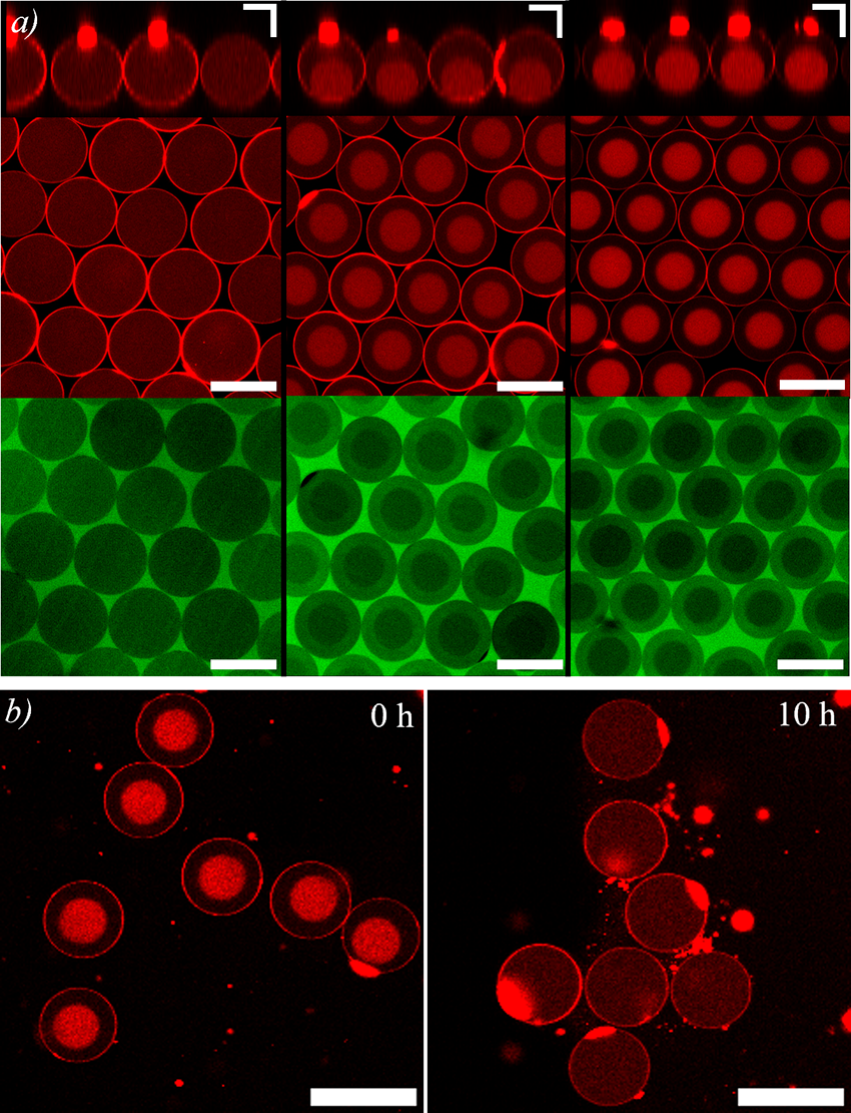
(a) Confocal fluorescence images of Pluronic vesicles encapsulating a mixture of 2.5% (w/v) PEG and 2.5% (w/v) dextran, collected in aqueous solutions of PEG at 7, 9, and 11% (w/v) from left to right. The top panel shows the vertical projection of the vesicles (scale bars are 25 μm), while the middle and bottom panels show their equatorial plane in both the dextran-Rh channel and the mPEG-FITC channel, respectively (scale bars are 50 μm). (b) Confocal fluorescence images showing the same vesicles, immediately after collection in an aqueous solution of 8.5% (w/v) PEG (left) and after thermodynamic equilibration of the sample (right). Scale bars are 25 μm.

The Pluronic membrane is additionally used in this study because it enables the transport of one of the encapsulated components of the ATPS, PEG (6 kDa), across the membrane. To demonstrate the permeation of PEG across the membrane, we include a fluorescently labeled PEG, mPEG-FITC (5 kDa), in the collection medium. We observe that mPEG-FITC penetrates into the vesicle, as shown in Movie S1, and upon equilibration partitions preferentially within the PEG-rich compartment, as shown in the bottom panel of Figure 2a. By contrast, a fluorescently labeled dextran, dextran-FITC, of the same molecular weight but a hydrodynamic radius about half that of the mPEG-FITC,^32^ is not capable of permeating the Pluronic membrane, as shown in Figure S2. This indicates that the permeability of this membrane is highly selective to the chemical nature of the polymer for molecules above a certain size, as summarized in Figure S2 and further detailed in Section S2 of the Supporting Information. To further demonstrate the permeation of the ATPS component, PEG (6 kDa), through the membrane, we encapsulate a mixture of 2.5% (w/v) dextran and 2.5% (w/v) PEG in vesicles and collect the vesicles in a 8.5% (w/v) PEG aqueous solution. We observe that upon collection the vesicles are phase separated due to the rapid outflow of water, as shown in the leftmost panel of Figure 2b, but a single, homogeneous phase is observed within the vesicles once the sample is fully equilibrated through the slower permeation of PEG, as shown in the rightmost panel of Figure 2b. Therefore, the different dynamics associated with water and PEG permeation provides a route to create out-of-equilibrium phase separations that evolve toward thermodynamic equilibrium by the slow permeation of the permeating component, which may ultimately reverse the phase separation within the vesicles, depending on the concentration of the permeating component in the outer aqueous phase. This is a hallmark of a responsive material, a cell-like structure capable of sensing the environment and consequently responding by transporting other molecules besides water.

The high encapsulation efficiency and high throughput of the microfluidic approach to vesicle production described above provide a versatile platform for the determination of phase diagrams of ATPSs within vesicles. For this, vesicles should be fully equilibrated, which implies the absence of net flows of both water and the permeating component. After equilibration, we determine the phase diagram of the described ATPS, experimentally measuring two quantities: the mean fluorescence intensity of each phase within compartmentalized vesicles using fluorescently labeled dextran and the osmolarity of homogeneous PEG/dextran mixtures at different ratios and total concentrations. The concentration of dextran in each phase is obtained from the mean fluorescence intensity of each compartment, measured on confocal fluorescence microscopy images, using the calibration shown in Figure S3. The same strategy could be applied to PEG, but the available fluorescently labeled PEG has a different molecular weight than the encapsulated PEG. Therefore, instead, the concentration of PEG in each phase is determined here from the equilibrium condition for the chemical potential of PEG in the phases coexisting within the vesicles and in the external phase: μ_1_^I^ = μ_1_^II^ = μ_1_^III^. Modeling the chemical potential of PEG as a virial expansion truncated at second order,^33−39^ this equilibrium condition can be written as
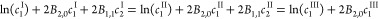
1where I, II, and III are the dextran-rich, the PEG-rich, and the collection phases, respectively, and 0, 1, and 2 are the water, PEG, and dextran molecules, respectively. *B*_2,0_ and *B*_1,1_ are virial coefficients that account for two-body solvent-mediated interactions between PEG–PEG and PEG–dextran molecules, respectively. The suitability of osmotic virial expansions to second order to solve liquid–liquid equilibria^40^ and the use of the PEG chemical potential as the basis for our approach are discussed in Section S4 of the Supporting Information (Figures S4–S6).^41^

To determine the virial coefficients, *B*_2,0_ and *B*_1,1_, we measure the osmolarity of aqueous solutions of PEG of different concentrations and that of homogeneous mixtures of PEG and dextran. The experimental data, shown by the solid symbols in Figure 3, are well-described by an osmotic second-order virial expansion
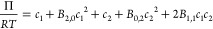
2where *B*_0,2_ is the virial coefficient for the two-body solvent-mediated interactions between dextran–dextran molecules. Fitting of the data of pure PEG solutions (black symbols) to eq 2 yields *B*_2,0_ = 0.224 ± 0.008 m^3^/mol, in agreement with the literature values.^42,43^ For the mixtures (color symbols), we obtain *B*_0,2_ = 5.4 ± 1.3 m^3^/mol, also in agreement with literature values,^44,45^ and *B*_1,1_ = 1.4 ± 0.2 m^3^/mol.

**Figure 3:**
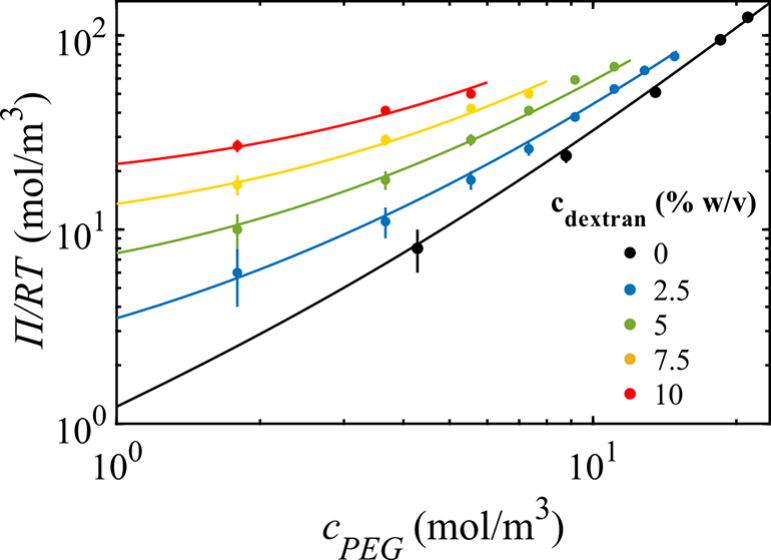
Osmotic pressure of different PEG/dextran mixtures as a function of PEG concentration (solid symbols) and fittings to the virial expansion in eq 2 (solid lines). The error bars are the standard deviations of four measurements.

We assume that the concentration of PEG in the collection medium, *c*_1_^III^, remains constant upon equilibration of the sample, acting as a source or sink for PEG molecules because the collection phase volume is very large compared to that of the encapsulated phases. Therefore, μ_1_^III^ is fixed by the external aqueous phase, and the PEG concentration in each phase within the vesicles is calculated as μ_1_^I^ = μ_1_^III^ for the dextran-rich core and μ_1_^II^ = μ_1_^III^ for the PEG-rich shell, using eq 1. Importantly, by fixing the concentration of PEG in the collection medium while varying the ratio of PEG to dextran in the encapsulated mixture, we obtain compartments with fixed composition, within the experimental uncertainty, but varying sizes, as exemplified by Figure 4a, which thus lie on a single tie-line. The tie-line slopes determined by collecting the vesicles in 10, 11 and 12% PEG, shown by the different colors in Figure 4b give an average slope of −0.26 ± 0.03, whereas the value reported for the bulk system is −0.55.^46,47^ This discrepancy is likely due to differences in the molecular weight and monodispersity of polymers from different suppliers, as detailed in Table S1, but also affected by the use of eq 1 to determine the PEG concentration instead of a direct measurement, as further discussed in Section S4 of the Supporting Information. The overall composition of the vesicles, shown by the solid symbols in Figure 4b, is then calculated as
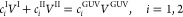
3where *V*^I^ and *V*^II^ are the volumes of the dextran-rich and PEG-rich compartments, respectively, and *V*^GUV^ is the volume of the whole vesicle, which are all calculated from the radii measured on confocal fluorescence microscopy images. Moreover, to determine the coexistence or binodal curve, we examine vesicles collected in aqueous solutions with lower PEG concentrations. The encapsulated mixtures that do not phase separate upon equilibration in the collection media are shown by the hollow symbols in Figure 4b. Fitting of these data to the binodal model,^48^ as further detailed in Section S4 of the Supporting Information, results in the dashed line shown in Figure 4b, which is in fair agreement with the tie-line determined in bulk, as shown in Figure S6.^46,47^ Similarly, when we encapsulate an aqueous solution containing solely 5% dextran within permeating vesicles and collect them in aqueous solutions with increasing PEG concentration, we do not observe coexistence of phases within the vesicles, as shown in Figure S7; the composition of the resultant vesicles lies below the binodal curve. Importantly, although the phase diagram of the ATPS is not affected by membrane confinement or permeability, the selective permeability implemented in these vesicles enables fine-tuning of the final ratio of components within the vesicles, which remains fixed when the system is confined in an impermeable vesicle, as further detailed in Section S6 of the Supporting Information (Figures S8–S10).

**Figure 4:**
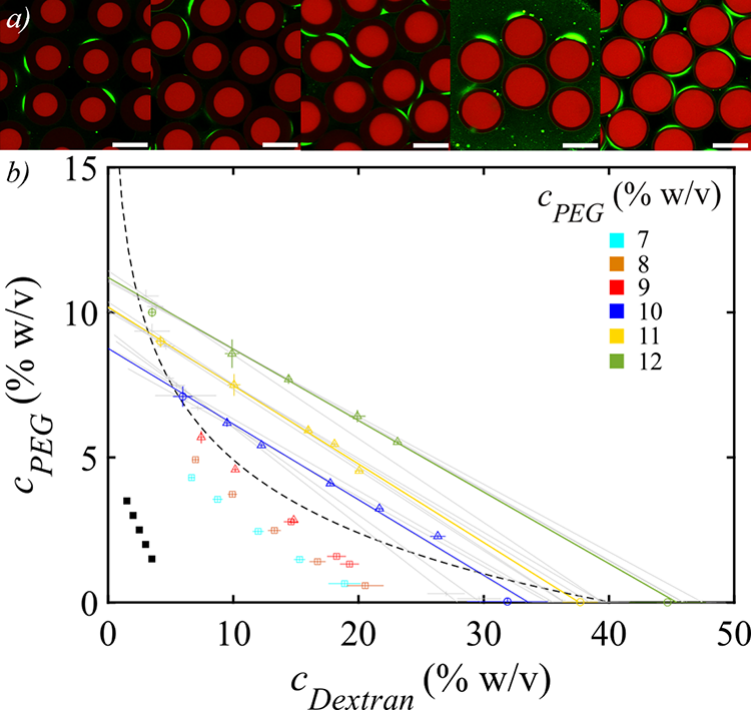
(a) Confocal fluorescence microscopy images showing vesicles encapsulating different PEG/dextran ratios, collected in a 10% (w/v) aqueous solution of PEG (blue tie-line). Dextran is fluorescently labeled with dextran-Rh (red channel), and the Pluronic L121 is fluorescently labeled with DPPE-NBD (green channel). Scale bars are 50 μm. (b) Phase diagram of the PEG/dextran system encapsulated within permeable vesicles (*N* ≈ 70). The composition of phases I and II are shown as circles; the overall composition of the vesicles as triangles in the two-phase region and as squares in the single-phase region; the initial encapsulated PEG/dextran mixtures as solid black squares. Average tie-lines are shown as solid color lines and individual tie-lines as thin gray lines. The dashed line is a fit of the experimental data to the binodal model in ref (48).

Although robotic pipetting stations significantly simplify the preparation and phase diagram determination of ATPSs,^49^ the use of microfluidic technologies to produce vesicles that efficiently encapsulate ATPSs provides a powerful platform to gain external control on the phase behavior of ATPSs. Because vesicle membranes, much like cell membranes, are permeable to water, encapsulated mixtures of dissimilar polymers can be concentrated up to the phase separation threshold by applying osmotic stress. Fluorescent labeling of the encapsulated polymers would then enable direct determination of the composition of each phase from fluorescence intensity measurements on confocal fluorescence microscopy images. By contrast, if only one of the polymers is fluorescently labeled but the second virial coefficients of the system are independently measured, the thermodynamic condition of equilibrium of chemical potential of the unlabeled polymer across the coexisting phases provides an alternative means to determine the composition of each phase of the ATPS. This second strategy to phase diagram determination is here demonstrated. Moreover, we show that membrane permeability provides an effective route to affect the dynamics of phase separation of the encapsulated system and tune the ratio of the encapsulated components. Importantly, this model system may be envision as a model cell nucleus, where the vesicle membrane mimics the selective permeability of the nuclear envelope, enabling transport of macromolecules, such as RNA and proteins, between the cytoplasm and the nucleoplasm, depending on external conditions, and where the phase-separated compartment mimics a model nucleolus that assembles or disassembles also depending on external conditions. In this context, the different dynamics associated with membrane permeation depending on molecule size and structure enable out-of-equilibrium phase separations that are spontaneously reversed over time through molecular transport, providing a route to instantly separate and then connect metabolic and signaling pathways.

## Supporting Information Available

Experimental procedures, complementary membrane permeability experiments, details on confocal microscope calibration for dextran concentration determination, discussion on the thermodynamics of ATPSs, complementary results on the encapsulation of pure dextran solutions within permeable vesicles and discussion on ATPS formation from homogeneous polymer mixtures within permeable and impermeable membranes (PDF)
Movie S1: demonstration of PEG permeation across the vesicle membrane during phase separation of the encapsulated polymer mixture (AVI)

## Supplementary Material

mz3c00341_si_001.pdf

mz3c00341_si_002.avi
