# Supplementary material for: Aqueous Two-Phase Systems within Selectively Permeable Vesicles
Source: ACS Macro Lett. 2023 Jul 27;12(8):1132–7. doi: 10.1021/acsmacrolett.3c00341 (PMC10433528; doi:10.1021/acsmacrolett.3c00341)
Supplement: Supplementary file 1 — mz3c00341_si_001.pdf [file mz3c00341_si_001.pdf]

Supporting Information for "Aqueous two-phase systems within selectively permeable vesicles"

Berta Tinao, Juan L. Aragones, and Laura R. Arriaga*

Department of Theoretical Condensed Matter Physics, Condensed Matter Physics Center (IFIMAC) and Instituto Nicolás Cabrera, Universidad Autónoma de Madrid, 28049, Madrid, Spain

E-mail: laura.rodriguezarriaga@uam.es

S1 Experimental Section

S1.1 Chemicals

Dextran from Leuconostoc spp. (70 kDa), polyethylene glycol (PEG, 6kDa), poly(vinyl alcohol) (PVA, 13–23 kDa, 87–89% hydrolyzed), Pluronic L121 and chloroform, are all purchased from Sigma. Hexane is purchased from VWR. 1,2-dioleoyl-sn-glycero-3-phosphoethanolamine-N-(lissamine rhodamine B sulfonyl) (ammonium salt) (DOPE-Rh) and 1,2-dipalmitoyl-sn-glycero-3-phosphoethanolamine-N-(7-nitro-2-1,3-benzoxadiazol-4-yl) (DPPE-NBD), purchased from Avanti Polar Lipids, are used to label the vesicle membrane. Rhodamine B isothiocyanate–dextran (Dextran-Rh, 70 kDa) and fluorescein isothiocyanate–dextran (Dextran-FITC, 5 kDa), both purchased from Sigma, and fluorescein isothiocyanate methoxy-PEG (FITC-mPEG, 5 kDa), purchased from Genochem, are used to label the dextran-rich phase and to study PEG permeation, respectively. Milli-Q water from a Millipore system (resistivity 18.2 MΩ·cm) is used to prepare the aqueous phases.

S1.2 Fabrication and Operation of the glass-capillary device

A 40 mm long square glass capillary (CM Scientific, ID 1.00 x 1.00 mm) is attached onto a glass microscope slide using 5 minute epoxy resin. A 80 mm cylindrical capillary (OD 1.00 mm; ID 0.58 mm, World Precision Instruments, Inc.) is pulled using a micropipette puller (P-2000 Sutter Instrument, Inc.) to yield two tapered cylindrical capillaries, one to be used as injection capillary and the other as collection capillary. Both capillary tips are carefully sanded (sand paper, 2000 grit) till a final diameter of 60-80 µm and 120-160 µm, for the injection and collection capillary, respectively. The walls of the injection capillary are rendered hydrophobic by immersing the tip of the capillary in n-octadecyl-trimethoxysilane (Sigma) for 20 minutes, washed with chloroform and dried. These tapered cylindrical capillaries are inserted at the ends of the square capillary and axially aligned under an inverted microscope, fixing the distance between both tips to 60-80 µm. Once aligned, they are fixed to the glass slide with epoxy. An additional smaller capillary pulled into a L-shape using a flame is inserted into the injection capillary and again fixed to the slide with epoxy. Finally, we place three dispensing needles (Gauge 21) on top of the three junctions connecting the capillaries and glue them to the slide with epoxy. A photo of the glass-capillary device is shown in the bottom panel of Figure S1. The device inlets are connected with polyethylene tubing (ID 0.86 mm; Warner Instruments, Inc.) to syringes, each placed on a flow-rate controlled pump (New Era Pump Systems, Inc.), containing the inner, middle and outer phases (plastic syringes from Terumo for aqueous phases and Hamilton glass syringe for the oil phase), as schematically shown in the bottom panel of Figure S1. These phases are infused at typical flow rates of 0.5 mL/h, for the inner and middle phases, and 3 mL/h, for the outer phase. The inner water phase, which consists of a homogeneous polymer mixture of polyethylene glycol (PEG, 6 kDa) and dextran (70 kDa), at a total polymer concentration of 5% (w/v; mass of solute per volume of solution) and varying PEG/dextran ratio, is injected through the smaller capillary on the left. The middle oil phase, which contains 20% (w/v) Pluronic L121 (PEG-PPG-PEG, 4.4 kDa) dissolved in

a mixture of 36% (v/v) chloroform and 64% (v/v) hexane, is injected through the injection capillary. The simultaneous injection of the inner and middle phases within the injection capillary results in the formation of a train of W/O droplets because, prior to device assembly, the walls of the injection capillary are rendered hydrophobic to favor contact of the oil phase with the capillary wall. The outer phase, a 10% (w/v) polyvinyl alcohol (PVA, 13-23 kDa) aqueous solution, is injected through the interstices between the injection capillary and the rectangular capillary on the left to encounter the large W/O droplets at the injection capillary tip, as schematically illustrated in the top panel of Figure S1. PVA provides the outer aqueous phase with the required viscosity to overcome the interfacial tension holding the drop at the tip, which ultimately results in re-emulsification of the W/O droplets into W/O/W double emulsion drops with ultrathin middle oil layers.[1,2] This operation regime is known as discontinuos dripping regime,[1] becuase it results in the alternative production of single and double emulsion drops, which are easily separated upon collection due to their different densities. When these drops are collected into vials containing a large volume of an aqueous solution of PEG (6 kDa) at concentrations ranging from 7 to 12% (w/v), double emulsions sink, facilitating their visualization on an inverted microscope, and their separation from the single emulsion drops that remain at the top of the vial.

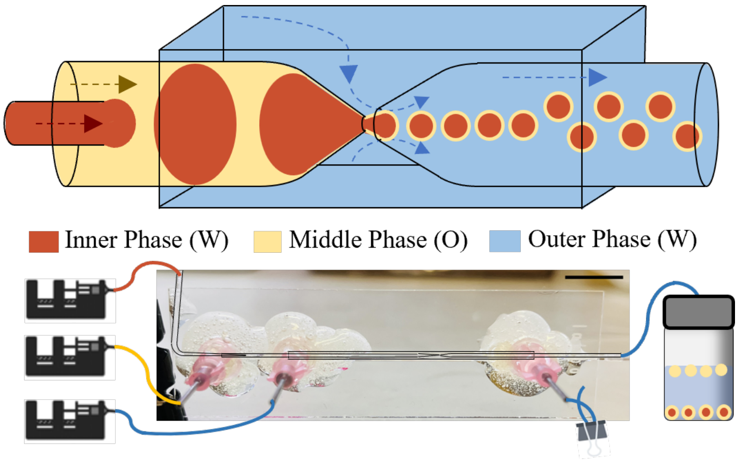

Figure S1: Schematic illustration of the glass-capillary microfluidic device (top) and exprerimental setup (bottom) used to produce W/O/W double emulsion drops with ultrathin shells. Scale bar in the device photograph is 1 cm.

S1.3 Osmolality

We measure the osmolality of aqueous solutions of PEG (6 kDa) and homogeneous aqueous mixtures of PEG and dextran at varying polymer ratios and total concentrations, using a freezing point osmometer (Osmomat 030, Gonotech GmbH).

S1.4 Imaging

We monitor the microfluidic production using an inverted optical microscope (VWR, VisiScope) equipped with a 10x air objective and a CCD camera (FLIR Blackfly 291). Compartmentalised vesicles are imaged using an inverted confocal fluorescence microscope (Nikon Eclipse Ti) with a 4-laser module (C2Si) and a 20x Plan Apo Air objective. Images are obtained in two different channels: the excitation wavelength for FITC-mPEGs is 488 nm and for dextran-Rh is 561 nm. The emission is collected using band pass filters ranging 500 - 560 nm for FITC and 570 - 1000 nm for Rh.

S2 Pluronic L121 membrane permeability

To study the permeability of the Pluronic membrane to different ions, molecules and macromolecules, we encapsulate an aqueous solution containing 5% dextran (70 kDa) within the vesicles and collect them in aqueous solution of different solutes. For solutes that are not fluorescently labeled, such as sodium and potassium ions or sucrose, we acquire bright field micrographs immediately after immersing the vesicles in the collection medium and 24 hours later, once the sample is equilibrated. We observe that in these three cases, vesicles swell despite being collected in media with a higher osmolarity than that of the encapsulated inner solution, as shown in Figure S2a-c; this indicates that these ions and sucrose are capable of permeating the membrane, thus increasing the osmolarity of the encapsulated aqueous phase. We also test the permeability of the Pluronic membrane to larger molecules using fluorescently labeled compounds. We first show that sulforhodamine, a hydrophilic dye of 558.7 g/mol, rapidly permeates the membrane, fluorescently labeling the inner water cores

of the vesicles, as shown in Figure S2d. Similarly, a fluorescently labeled protein, casein-FITC of 2062 g/mol, permeates the membrane and fluorescently labels the inner cores of the vesicles, as shown in Figure S2e. This data indicate that the Pluronic membrane is permeable to ions and molecules up to about 2 kDa. For larger macromolecules, the membrane exhibits a selective permeability for the chemical structure of PEG, because the Pluronic polymer comprising the membrane is made of PEG and PPG, which are of the same chemical nature as the permeating solute, as discussed in the main text. To demonstrate this selective permeability, we encapsulate within the vesicles an aqueous solution containing 5% dextran (70 kDa), like in the rest of the experiments, but also 0.5% dextran-Rh (70 kDa) and collect them in an aqueous solution of 0.5% dextran-FITC (5 kDa). After equilibration of the sample, dextran-Rh remains efficiently encapsulated within the vesicle cores, whereas dextran-FITC remains in the external medium, as shown in the confocal microscope image and quantified by the fluorescence intensity profile in Figure S2f; this indicates that the Pluronic membrane is impermeable to dextran, independently on dextran molecular weight, in the range between 5 to 70 kDa. The very different chemical structures of dextran and PEG that justify their different permeation capacities are included in Figure S2g. Dextran is a branched polyssacharide composed by glucose monomers, whereas PEG is a linear polymer. Although the hydrodynamic size in water for a PEG molecule is approximately double than that of a dextran molecule of comparable molecular weight,[3] penetration through the Pluronic membrane imposes a change in solvent quality, maintaining the dextran molecule in the external aqueous phase.

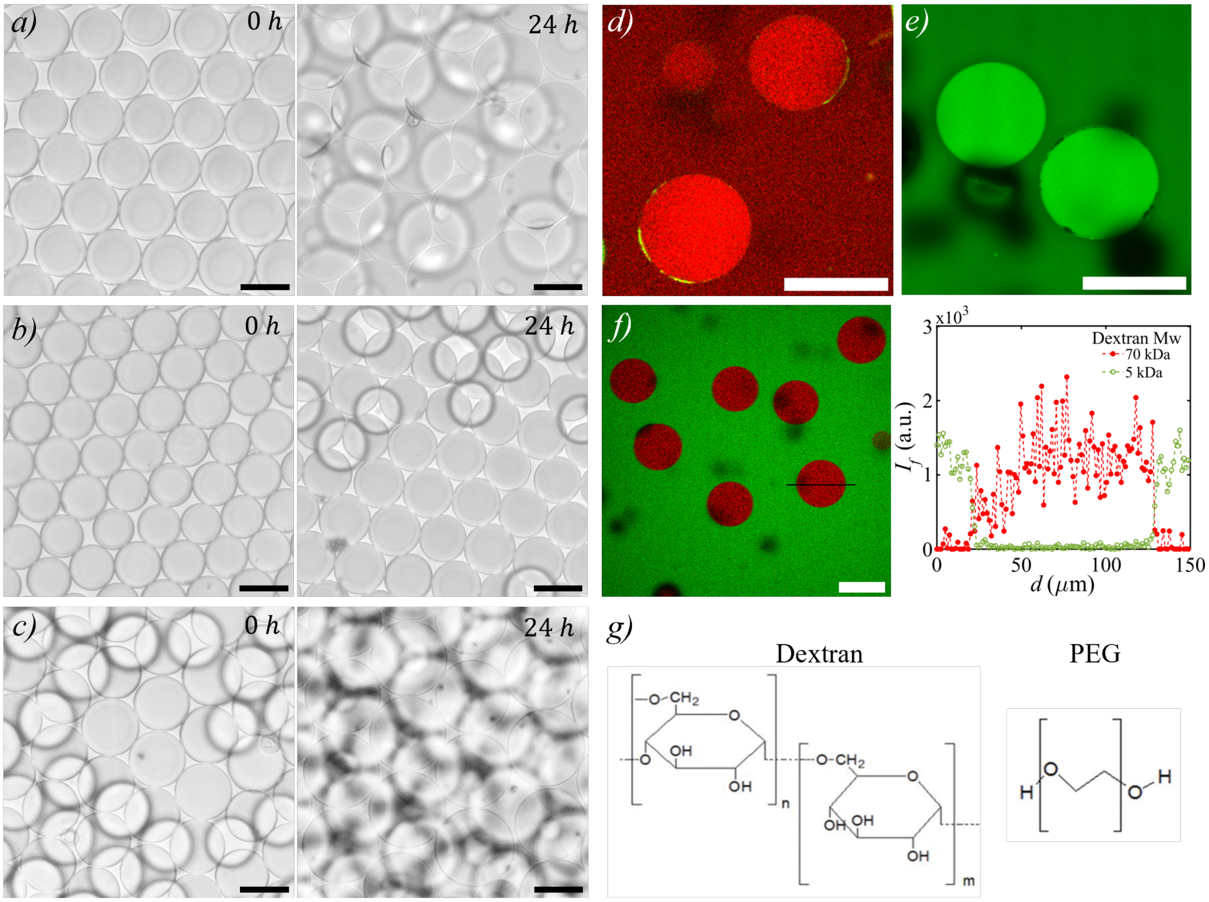

Figure S2: Microscope images showing Pluronic L121 vesicles encapsulating a 5% dextran solution and collected in different media: a) 280 mM NaCl solution; b) 280 mM KCl solution; c) 280 mM sucrose solution; d) 1 µM sulforhodamine solution; e) 0.1 mg/mL casein-FITC solution; f) 0.5% dextran-FITC (5 kDa) solution. Bright field microscopy images acquired immediately after and 24 h after collection are included to show vesicle swelling (scale bars are 50 µm). For fluorescently labeled collection media (d-e) we show confocal microscope images of the vesicles shortly after collection (scale bars are 100 µm). f) Overlayed confocal microscope image showing the fluorescence of dextran-Rh (70 kDa, red channel) encapsulated within the vesicles and dextran-FITC (5 kDa, green channel) in the collection medium, and quantification of the fluorescence intensity profile across the black line drawn on a single vesicle for the two channels. g) Chemical structure of dextran and PEG.

S3 Determination of dextran concentration

To determine the dextran concentration in each phase, we need to calibrate our confocal fluorescence microscope. For this, we prepare aqueous solutions of dextran with increasing concentration ranging from 5 to 30 % (w/v) and pour them into microscope chambers,

consisting of two cover slips separated by vacuum grease in a sandwich configuration. Typical images of the solutions are shown in Figure S3a, for a fixed laser power and a fixed gain. Complementarily, we acquire the same type of images while varying the gain. The mean fluorescence intensity of the whole image is then plotted against dextran concentration, which results in a calibration curve for each gain, as shown in Figure S3b. As expected, the mean fluorescence intensity of the image varies linearly with dextran concentration. Therefore, this calibration enables us to obtain the dextran concentration in each phase within the vesicle from the mean fluorescence intensity of each phase in the confocal microscope image.

To measure the mean fluorescence intensity of the dextran-rich phase, we focus at the equatorial plane of the inner dextran-rich core, as shown in the top panel of Figure S3c. To measure the mean fluorescence intensity of the PEG-rich phase, we focus at the equatorial plane of the whole vesicle, as shown in the bottom panel of Figure S3c, as this provides a larger area to average the fluorescence intensity for this phase because the dextran-rich phase is located at the bottom center of the vesicles. A typical distribution of dextran concentration within each phase is shown in the top panel of Figure S3d. The same images are used to measure the radius of both the dextran-rich phase and the whole vesicle, and a typical distribution of radius is shown in the bottom panel of Figure S3d.

The same procedure should be ideally applied to the PEG component to determine the PEG concentration in each phase directly. Unfortunately, we did not find any commercially available fluorescently-labeled PEG of the exact same molecular weight as the encapsulated PEG (6 kDa), this prevents us from using this direct strategy to phase diagram determination. Instead, we experimentally determine the virial interaction coefficients between similar and dissimilar molecules using freezing-point osmometry and then calculate the PEG concentrations in each phase using a thermodynamic model.

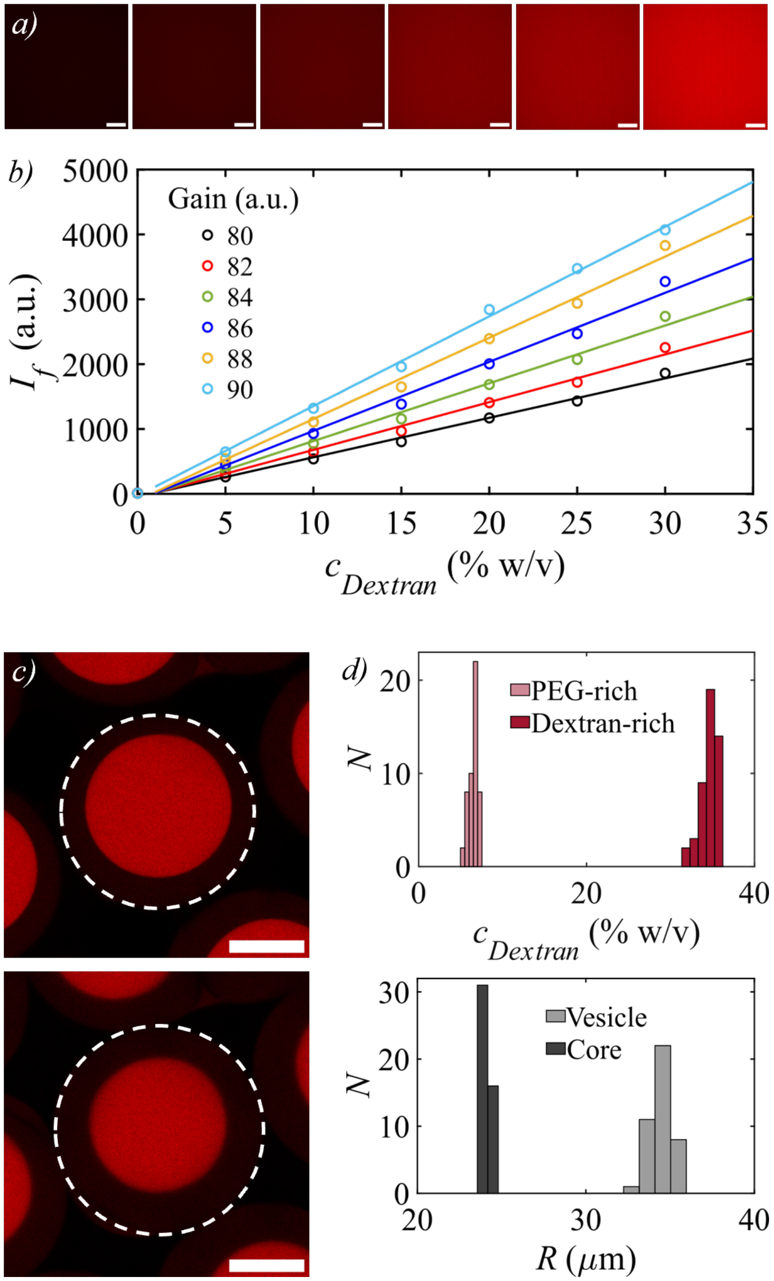

Figure S3: a) Representative confocal fluorescence microscope images obtained at a gain of 84 a.u. of aqueous solution with increasing concentration of dextran: 5, 10, 15, 20, 25 and 30 % (w/v) from left to right. b) Linear dependency of the mean fluorescence intensity of the solution on dextran concentration. c) Confocal fluorescence microscope image of a typical vesicle, encapsulating a mixture of 2.5% (w/v) PEG and 2.5% (w/v) dextran and collected in a 10 % (w/v) PEG solution, acquired at the equatorial plane of the dextran-rich phase (top) and the equatorial plane of the vesicle (bottom). The dashed white line illustrates membrane position. Scale bar is 25 μm. d) Distribution of dextran concentration across the phases (top) and radius of the dextran-rich core and vesicle (bottom).

S4 Thermodynamics of an ATPS encapsulated in vesicles

Much like in the bulk system, coexisting phases within the vesicles equilibrate the chemical potentials of all the species across the encapsulated phases:

$$\mu_i^I = \mu_i^{II} \quad i = 0, 1, 2 \tag{S1}$$

where the solvent is denoted by '0' and the two polymers, PEG and dextran, are denoted by '1' and '2', respectively; these conditions can be used to determine the concentration of the PEG component in each phase. However, this requires application of a theoretical model. In our work, we use virial expansions to second order, because they are thermodynamically consistent.[4] Within this framework, the chemical potentials of the different species can be written as:

$$\mu_1 = \mu_1^0 + RT[ln(c_1) + 2B_{2,0}c_1 + 2B_{1,1}c_2] \tag{S2}$$

$$\mu_2 = \mu_2^0 + RT[ln(c_2) + 2B_{0,2}c_2 + 2B_{1,1}c_1] \tag{S3}$$

$$\mu_0 = \mu_0^0 - RT[c_1 + c_2 + B_{2,0}(c_1)^2 + B_{0,2}(c_2)^2 + 2B_{1,1}c_1c_2] \tag{S4}$$

However, because this model only considers two-body solvent-mediated interactions between polymers, its application may be unrealistic for other systems. Here, we use it because this osmotic virial expansion to second order agrees fairly well with the experimental phase diagram determined for the PEG 3350 - dextran T70 system,[5] which is very close in molecular weight to the system under study. Moreover, to further validate this approach, we calculate the 'residual' terms, Δ,[4] for the chemical potential of dextran within the two phases encapsulated within the vesicles, Δ_2^I and Δ_2^{II}:

$$\Delta_2^I = ln(c_2)^I + 2B_{0,2}c_2^I + 2B_{1,1}c_1^I \tag{S5}$$

$$\Delta_2^{II} = ln(c_2)^{II} + 2B_{0,2}c_2^{II} + 2B_{1,1}c_1^{II} \tag{S6}$$

using the dextran concentrations independently obtained from fluorescence intensity measurements (Section S3) and the PEG concentrations obtained from $\mu_1^I = \mu_1^{II}$ with our measured virial coeficcients, as further detailed in the main text. The ratio between these residual terms should equal 1 if perfect agreement with the model is found. We find that the ratio between residual terms is of the order of 0.8, with the lower overall polymer concentrations giving a perfect agreement with the model, as shown in the top panel of Figure S4. Furthermore, we also calculate the residual terms for the chemical potential of water within the two phases encapsulated within the vesicles, Δ_0^I and Δ_0^{II}:

$$\Delta_0^I = c_1^I + c_2^I + B_{2,0}(c_1^I)^2 + B_{0,2}(c_2^I)^2 + 2B_{1,1}c_1^I c_2^I \tag{S7}$$

$$\Delta_0^{II} = c_1^{II} + c_2^{II} + B_{2,0}(c_1^{II})^2 + B_{0,2}(c_2^{II})^2 + 2B_{1,1}c_1^{II} c_2^{II} \tag{S8}$$

The ratio between these residual terms in the two encapsulated coexisting phases for water shows good agreement with the model only for the lower overall polymer concentrations in the vesicles, as shown in the bottom panel of Figure S4. The average ratio for larger overall concentrations is 0.6.

Alternatively, we calculate the phase diagram of the system using the solvent chemical potential as the basis for our approach. This alternative approach is justified because the experimental determination of the osmotic second virial coefficients that we use is based on the osmotic virial expansion of the solvent and this approach is expected to give considerable cancellation of higher order terms.[4] Therefore, we consider next the equilibration of the chemical potential of the water solvent between the external media and each of the coexisting phases with the vesicles to determine the PEG concentration in each phase. This approach provides negative values of PEG in the dextran-rich phase, as shown in Figure S5a, which is a physically unrealistic situation. However, considering that this means that there is a negligible PEG concentration in the dextran-rich phase and again solving the equilibrium

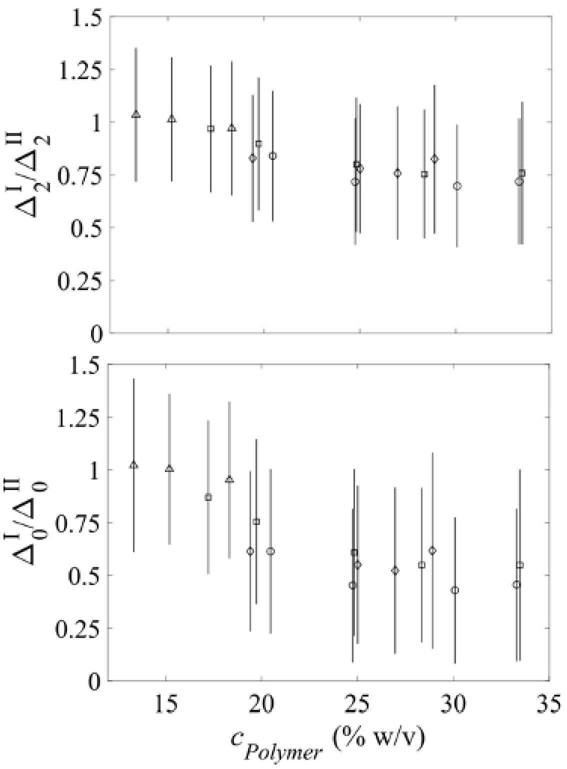

Figure S4: Ratio of the residual terms of the chemical potential of phase separated vesicles collected in 9 (triangles), 10 (squares), 11 (diamonds) and 12 (circles) % (w/v) PEG aqueous solutions for the dextran component (top) and for the water solvent (bottom) as a function of the overall polymer concentration in the vesicles.

condition for the chemical potential of water across the phases, we fairly recover the predicted phase diagram from equilibration of the chemical potential of PEG, as shown in Figure S5b. Therefore, we use the chemical potential of PEG as the basis of our approach.

The tie-line slopes determined from equilibration of the chemical potential of PEG across the phases give a mean value of (-0.26 ± 0.03), whereas those determined from equilibration of the water chemical potential give a mean value of (-0.35 ± 0.01), due to the unrealistic values of PEG concentration in the dextran-rich phase. The experimentally measured tie-line slope for this system in bulk is -0.55.[6] Although this discrepancy can be due to the different molecular weight and polydispersity of the polymers provided by different suppliers, we test if the use of the second virial expansion model underestimates the tie-line slope. For this, we use the concentrations of dextran in each phase, reported in literature for the bulk system, and predict the PEG concentration in each phase using the described models. We

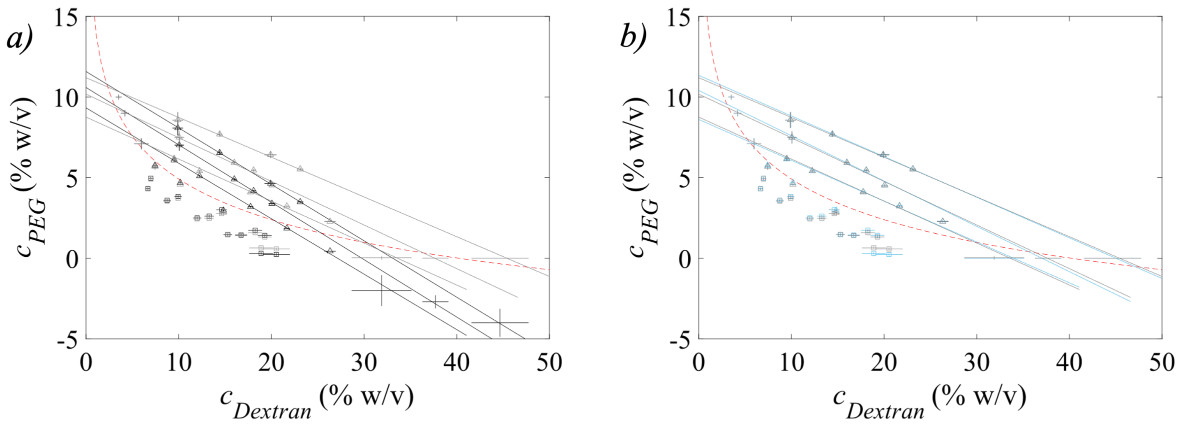

Figure S5: Phase diagrams obtained from the equilibration of the water solvent chemical potential across the vesicle membrane for the dextran concentrations in each phase determined from fluorescence intensity measurements: a) allowing the PEG concentration in each phase to obtain any value (black symbols and tie-lines) or b) forcing the PEG concentration in the dextran-rich phase to 0 (blue symbols and tie-lines). The light gray symbols and lines correspond to the phase diagram determined from equilibration of the PEG chemical potential (Figure 4 in the main text).

obtain that the predicted tie-line slopes vary from -0.41 to -0.37 depending on the species considered for the equilibration. This indicates that the remaining discrepancy with the reported bulk measurement may be arising from the differences in the polymers used, which are shown in Table S1.

Table S1: Comparison of the PEG (6 kDa) - dextran (70 kDa) systems in literature

Polymer	Supplier	Product	Lot/Batch	M_w (g/mol)	M_n (g/mol)	Ref.
PEG-6	Serva Fine Biochem.	-	463-80	6,000	-	[6]
dex-70	Minmedprom	-	680480	57,200	28,700	[6]
PEG-6	Aldrich	81260	BCBW6004	-	5,905	*
dex-70	Sigma	31390	BCBV0724	67,024	-	*

The composition of each coexisting phase within the vesicles defines the binodal curve separating the single-phase from the two-phase region of the phase diagram. We fit these compositions to a binodal model proposed in literature, which describes the concentration of one solute component as a continuous function of the other component in the binary system:[7]

$$ln(< V^* >_{210} \frac{w_2}{< M_{rms} >_2}) + < V^* >_{210} \frac{w_1}{< M_{rms} >_1} = 0 \qquad \text{(S9)}$$

where $< V^* >_{210}$ is the 'effective excluded volume', which can be used as the single model parameter, $< M_{rms} >_i$ is the geometrical average of the number- and weight-average molecular weights of each component, $< M_{rms} >= \sqrt{< M_w >< M_n >}$, and w_i are the concentrations in weight fraction of each component. The discontinuous dashed line in Figure S6 represents fitting of our data, shown by the triangles, to Eq. S9, which yields $< V^* >_{210} = (169 \pm 18)$ kg/mol. In this fitting, we use $< M_{rms} >_1 = < M_n >_1 = 5.905$ kg/mol and $< M_{rms} >_2 = < M_w >_2 = 67.024$ kg/mol, becuase these are the only reported molecular weights. We conclude that this value is reasonable by comparing it to that of the PEG3350 - Dex T70, which is reported to be 123 kg/mol.[4] Therefore, we include the fitting of our data to the binodal model in Figure 4 of the main text. Additionally, our binodal curve shows fair agreement with that reported for the same system measured in bulk,[6] shown by the squares in Figure S6, which provides a value of $< V^* >_{210} = (157 \pm 4)$ kg/mol upon fitting of the data to the binodal model, using $< M_{rms} >_1 = < M_w >_1 = 6.000$ kg/mol and $< M_{rms} >_2 = \sqrt{< M_w >_2 < M_n >_2} = 40.517$ kg/mol. Again we attribute the discrepancies between the determined binodal curves to the molecular weight and polydispersity of polymers from different suppliers, summarized in Table S1.

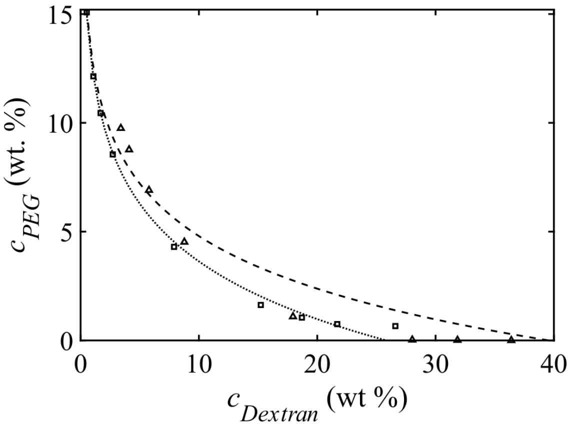

Figure S6: Fitting of the experimental points on the binodal curve determined in this work (triangles) and those previously reported by bulk methods (squares; taken from Ref.[6]) to the binodal model. The dashed line corresponds to fitting of the triangles, and the dotted line to fitting of the squares.

We do not anticipate that other approaches based on the design of vesicles with enhanced

interactions between the membrane and one of the encapsulated phases could change drastically the determined phase diagram. Although the relative positioning and wetting angle between the membrane and the encapsulated phases can be affected by varying the interaction potentials at the interfaces,[8,9] the phase diagram of the system is largely dominated by the bulk phases. A change in interfacial tension of about 0.5 mN/m,[10] which is a reasonable value for a dextran-rich/PEG-rich interface, results in a typical Laplace pressure of about 10 Pa, while osmotic pressures in the system are of the order of several hundreds of kPa.

S5 Dextran-filled vesicles

Vesicles encapsulating solely the non-permeating component, dextran, do not exhibit a phase separation in the vesicle core independently on dextran concentration, as shown in Figure S7a. We calculate the dextran concentration either directly from fluorescence intensity measurements, performed on the confocal fluorescence microscopy images shown in Figure S7a or from the decrease in vesicle volume, using Eq. S10, measuring the vesicle radii on the same confocal microscopy images.

$$c_2^{GUV,i} V_2^{GUV,i} = c_2^{GUV,f} V_2^{GUV,f} \tag{S10}$$

where $c_2^{GUV,i}$ is the overall polymer concentration initially encapsulated within a vesicle of volume $V_2^{GUV,i}$, and $c_2^{GUV,f}$ is the final dextran concentration in the vesicle due to the reduction of the vesicle volume to a value $V_2^{GUV,f}$. The correlation between both calculations gives a slope of (1.19 ± 0.03). The amount of PEG inside the vesicle should be that required to equilibrate the chemical potential of PEG across the membrane. For example, for a PEG concentration in the collection medium of 12.5% (w/v), we obtain a dextran-rich phase with 50% (w/v) dextran and a negligible PEG concentration, using the second order virial expansion for PEG equilibration, described in Section S4. A single, homogeneous phase is then obtained in all of these vesicles.

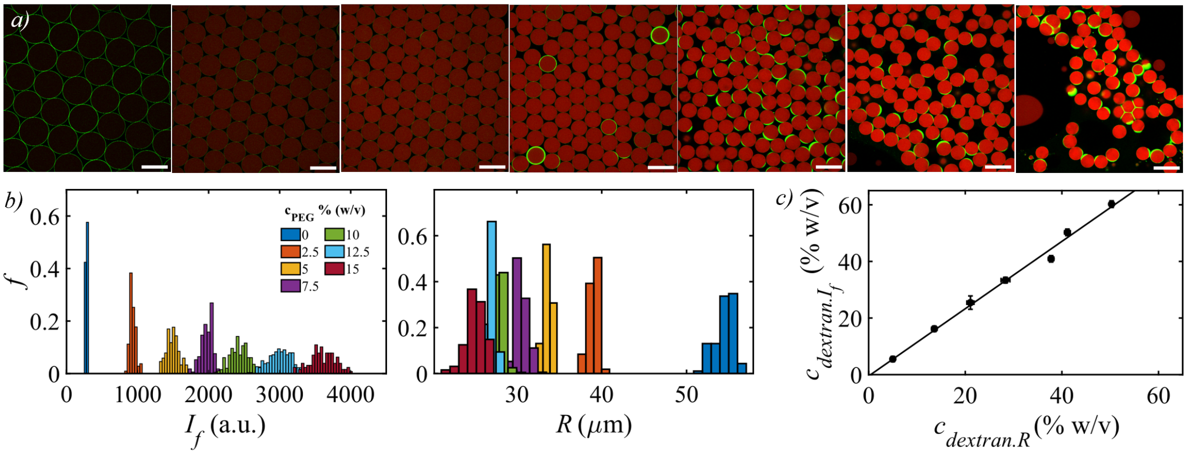

Figure S7: a) Confocal fluorescence microscope images showing vesicles encapsulating an initial concentration of dextran of 5% (w/v), collected in PEG solutions of 0, 2.5, 5, 7.5, 10, 12.5 and 15% (w/v), from left to right. Vesicle membrane is fluorescently labeled with DPPE-NBD. Scale bars are 100 um. b) Distributions of mean fluorescence intensity (left panel) and vesicle radius (right panel) for the images shown in a). c) Correlation between the dextran concentration obtained from fluorescence intensity measurements and that obtained from the vesicle radius.

S6 ATPS formation within vesicles

Although, once thermodynamic equilibrium is achieved, the encapsulated system organizes as two coexisting phases with the same composition than in a bulk system, the presence of a membrane provides external control on the overall polymer concentration within the vesicles. Inspired by cell membrane permeability, we address here the differences between encapsulating the system within a membrane solely permeable to water (impermeable vesicle) or within a membrane that additionally enables permeation of one of the solute components of the ATPS (permeable vesicle). An aqueous mixture of two polymers encapsulated by an impermeable membrane leads to an ATPS, upon concentration of the mixture by application of an osmotic stress. In this case, the PEG component in the external phase acts as an osmolyte, illustrated as green dots in the left part of Figure S8. The presence of this osmolyte in the external phase results in a water outflow that makes the vesicle shrink until the osmotic pressure across the membrane is equilibrated. Importantly, the water outflow preserves the

ratio of the two encapsulated polymers. This condition can be written as,

$$c_1^f/c_2^f = c_1^i/c_2^i \tag{S11}$$

where c_1^i and c_2^i are the initial concentrations of polymer 1 and 2, encapsulated in the vesicles and c_1^f and c_2^f are their final concentrations achieved through a water outflow within the impermeable vesicles. Therefore, a homogeneous mixture of polymers encapsulated within an impermeable membrane crosses the binodal curve to the two-phase region following a straight line of slope 1, as illustrated schematically by the path A to B in Figure S8. By contrast, when the vesicle membrane is additionally permeable to one of the encapsulated polymers, the PEG component is no longer an osmolyte becuase, much like the water solvent, it can freely permeate the membrane, as illustrated by the continuous green colour in the external phase in the right part of Figure S8. The differences in osmotic pressure or chemical potential across the membrane in this case are equilibrated by both water and PEG outflows. To confirm the net outflow of PEG, we calculate the ratio of the encapsulated polymers upon equilibration of the mixture through the selectively permeable membrane implemented in this study, from the overall concentrations shown in Figure 4, and compare it with the initially encapsulated ratio of polymers. Our results show that the ratio of PEG to dextran upon equilibration of the encapsulated mixture is always smaller than the ratio initially encapsulated, as shown in Figure S9. Therefore, we conclude that, although the thermodynamics of the system remains unchanged by membrane permeability, the implementation of a selectively permeable membrane provides an alternative route to explore the system. For example, moving from A to C, as illustrated schematically in Figure S8, is not possible through an impermeable membrane; to achieve C with an impermeable membrane, a different initial ratio of the polymers, exemplified by A' in Figure S8, needs to be used. The route provided by our selective permeability to explore the phase diagram of this system is summarized in Figure S10, where the different colors show the different paths followed by

each initial composition upon increasing PEG concentration in the external media; this may be important to understand phase separations within the cell cytoplasm or nucleoplasm as cell membranes are generally selectively permeable to other molecules besides water.

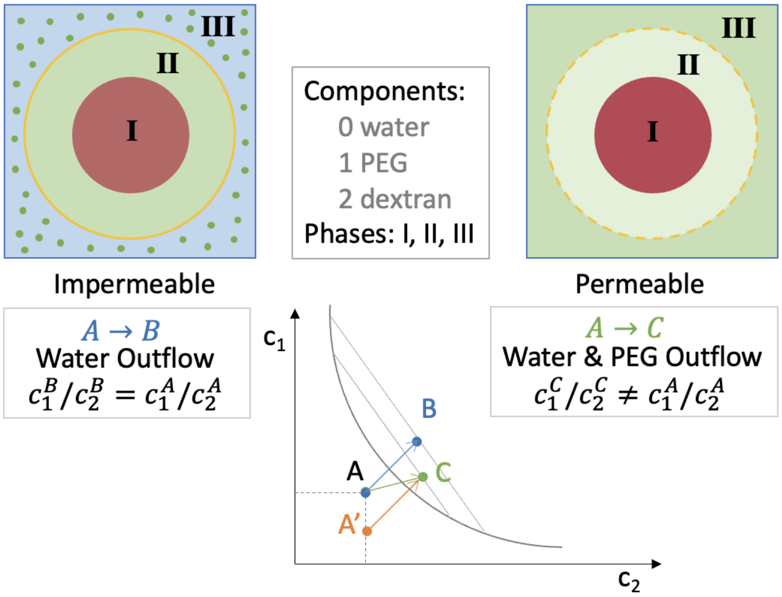

Figure S8: Schematic illustration of a compartmentalized vesicle with an impermeable (left) a selectively permeable membrane (right). The formation of the ATPS from a homogeneous mixture of composition A follows different routes for impermeable (A→B) and permeable membranes (A→C); arriving to C in an impermeable membrane requires encapsulating a composition A'.

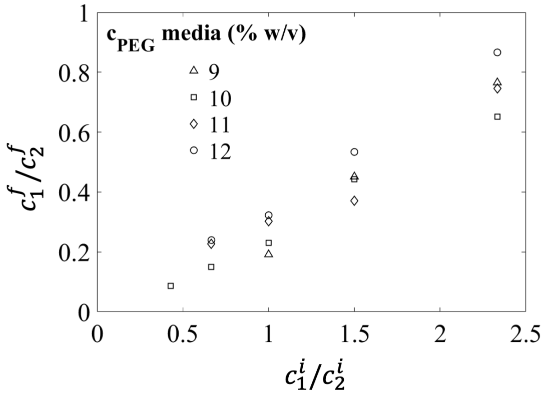

Figure S9: Ratio of PEG to dextran concentration as a function of the initial ratio of PEG to dextran concentration encapsulated within selectively permeable vesicles.

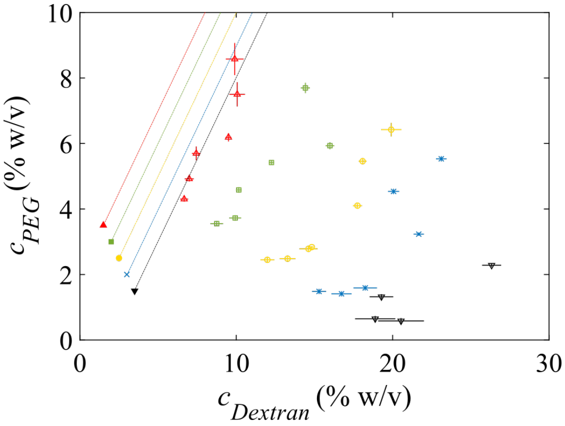

Figure S10: Equilibrated PEG and dextran concentrations within PEG permeable vesicles upon icreasing PEG concentration in the external medium for different intial ratios of PEG-to-dextran, indicated by the different colors. The dashed lines are the expected quilibrium PEG and dextran concentrations within impermeable vesicles.

Supplementary Movie S1

The movie shows the fluorescence of mPEG-FITC on the leftmost panel (green channel), that of dextran-Rh in the middle panel (red channel) and the overlay of both channels in the rightmost panel. The Movie shows the evolution of the vesicles during a time course of 25 min 45 s. The scale bar is 50 µm.

References

(1) Kim, S.-H.; Kim, J. W.; Cho, J.-C.; Weitz, D. A. Double-emulsion drops with ultra-thin shells for capsule templates. Lab on a Chip **2011**, 11, 3162–3166.

(2) Arriaga, L. R.; Datta, S. S.; Kim, S.-H.; Amstad, E.; Kodger, T. E.; Monroy, F.; Weitz, D. A. Ultrathin shell double emulsion templated giant unilamellar lipid vesicles with controlled microdomain formation. Small **2014**, 10, 950–956.

(3) Armstrong, J.; Wenby, R.; Meiselman, H.; Fisher, T. The Hydrodynamic Radii of Macromolecules and Their Effect on Red Blood Cell Aggregation. Biophysical Journal **2004**, 87, 4259–4270.

(4) Guan, Y.; Lilley, T. H.; Treffry, T. E. Theory of phase equilibria for multicomponent aqueous solutions: Applications to aqueous polymer two-phase systems. Journal of the Chemical Society, Faraday Transactions **1993**, 89, 4283–4298.

(5) Haynes, C.; Beynon, R.; King, R.; Blanch, H.; Prausnitz, J. Thermodynamic properties of aqueous polymer solutions: poly (ethylene glycol)/dextran. The Journal of Physical Chemistry **1989**, 93, 5612–5617.

(6) Zaslavsky, B. Y.; Borvskaya, A. A.; Gulaeva, N. D.; Miheeva, L. M. Physico-chemical features of solvent media in the phases of aqueous polymer two-phase systems. Biotechnology and bioengineering **1992**, 40, 1–7.

(7) Guan, Y.; Lilley, T. H.; Treffry, T. E. A new excluded volume theory and its application to the coexistence curves of aqueous polymer two-phase systems. Macromolecules **1993**, 26, 3971–3979.

(8) Long, M. S.; Cans, A.-S.; Keating, C. D. Budding and Asymmetric Protein Microcompartmentation in Giant Vesicles Containing Two Aqueous Phases. Journal of the American Chemical Society **2008**, 130, 756–762, PMID: 18092782.

(9) Li, Y.; Lipowsky, R.; Dimova, R. Transition from Complete to Partial Wetting within Membrane Compartments. Journal of the American Chemical Society **2008**, 130, 12252–12253, PMID: 18712871.

(10) Liu, Y.; Lipowsky, R.; Dimova, R. Concentration dependence of the interfacial tension for aqueous two-phase polymer solutions of dextran and polyethylene glycol. Langmuir **2012**, 28, 3831–3839.
